# Human electromagnetic and haemodynamic networks systematically converge in unimodal cortex and diverge in transmodal cortex

**DOI:** 10.1371/journal.pbio.3001735

**Published:** 2022-08-01

**Authors:** Golia Shafiei, Sylvain Baillet, Bratislav Misic

**Affiliations:** McConnell Brain Imaging Centre, Montréal Neurological Institute, McGill University, Montréal, Canada; University of Glasgow, UNITED KINGDOM

## Abstract

Whole-brain neural communication is typically estimated from statistical associations among electromagnetic or haemodynamic time-series. The relationship between functional network architectures recovered from these 2 types of neural activity remains unknown. Here, we map electromagnetic networks (measured using magnetoencephalography (MEG)) to haemodynamic networks (measured using functional magnetic resonance imaging (fMRI)). We find that the relationship between the 2 modalities is regionally heterogeneous and systematically follows the cortical hierarchy, with close correspondence in unimodal cortex and poor correspondence in transmodal cortex. Comparison with the BigBrain histological atlas reveals that electromagnetic–haemodynamic coupling is driven by laminar differentiation and neuron density, suggesting that the mapping between the 2 modalities can be explained by cytoarchitectural variation. Importantly, haemodynamic connectivity cannot be explained by electromagnetic activity in a single frequency band, but rather arises from the mixing of multiple neurophysiological rhythms. Correspondence between the two is largely driven by MEG functional connectivity at the beta (15 to 29 Hz) frequency band. Collectively, these findings demonstrate highly organized but only partly overlapping patterns of connectivity in MEG and fMRI functional networks, opening fundamentally new avenues for studying the relationship between cortical microarchitecture and multimodal connectivity patterns.

## Abstract 2

What is the relationship between functional network architectures inferred from electromagnetic and haemodynamic data? This study shows that superposition of electromagnetic networks at canonical frequency bands manifests as highly structured patterns of haemodynamic functional connectivity in the human brain, reflecting systematic variation in cytoarchitecture.

## Introduction

The structural wiring of the brain imparts a distinct signature on neuronal coactivation patterns. Interregional projections promote signaling and synchrony among distant neuronal populations, giving rise to coherent neural dynamics, measured as regional time series of electromagnetic or haemodynamic neural activity [1]. Systematic coactivation among pairs of regions can be used to map functional connectivity (FC) networks. Over the past decade, these dynamics are increasingly recorded without task instruction or stimulation; the resulting “intrinsic” FC is thought to reflect spontaneous neural activity.

The macroscale functional architecture of the brain is commonly inferred from electromagnetic or haemodynamic activity. The former can be measured using electroencephalography (EEG) or magnetoencephalography (MEG), while the latter is measured using functional magnetic resonance imaging (fMRI). Numerous studies—using both MEG and fMRI—have reported evidence of intrinsic functional patterns that are highly organized [2–9], reproducible [10–13], and comparable to task-driven coactivation patterns [12,14,15].

How do electromagnetic and haemodynamic networks relate to one another? Although both modalities attempt to capture the same underlying biological process (neural activity), they are sensitive to different physiological mechanisms and ultimately reflect neural activity at fundamentally different timescales [16–20]. Emerging theories emphasize a hierarchy of time scales of intrinsic fluctuations across the cortex [21–24], where unimodal cortex is more sensitive to immediate changes in the sensory environment, while transmodal cortex is more sensitive to prior context [25–30]. This raises the possibility that the alignment between the relatively slower functional architecture captured by fMRI and faster functional architecture captured by MEG may systematically vary across the cortex.

Previous reports have found some, but not complete, global overlap between the 2 modalities. Multiple MEG- and fMRI-independent components—representing spatiotemporal signatures of resting-state intrinsic networks—show similar spatial topography, particularly the visual, somatomotor, and default mode components [6–8,31]. The spatial overlap between large-scale networks has also been reported in task-based studies and with networks recovered from other modalities, such as EEG and intracranial EEG [32–36]. Moreover, fMRI and MEG/EEG yield comparable fingerprinting accuracy, suggesting that they encode common information [37–40]. Finally, global edge-wise comparisons between fMRI networks and electrocorticography (ECoG) [41], EEG [42–44], and MEG [45–47] also yield moderate correlations. Although global comparisons are more common when different modalities are studied, regional and network-level relationships have also been explored using electrophysiological and intracranial EGG recordings [36,48,49] as well as EEG and MEG recordings [45,50,51]. Regional comparisons of electrophysiological and fMRI recordings also suggest that the relationship between the two may be affected by distinct cytoarchitecture and laminar structure of brain regions, particularly in visual and frontal cortex [52–59]. How the coupling between fMRI and MEG connectivity profiles varies from region to region, and how this coupling reflects cytoarchitecture, is still not fully understood. Furthermore, previous studies have mostly assessed the association between haemodynamic and electromagnetic networks for separate frequency bands, investigating independent contributions of individual rhythms to haemodynamic connectivity. This effectively precludes the possibility that superposition and mixing of elementary electromagnetic rhythms manifests as patterns of haemodynamic connectivity [45,47,61].

How regional connectivity profiles of MEG and fMRI functional networks are associated across the cortex, and how their correspondence relates to the underlying cytoarchitecture, remains an exciting open question. Here, we use a linear multifactor model that allows to represent the haemodynamic FC profile of a given brain region as a linear combination of its electromagnetic FC in multiple frequency bands. We then explore how the 2 modalities align across the neocortex and investigate the contribution of cytoarchitectonic variations to their alignment.

## Results

Data were derived using task-free MEG and fMRI recordings in the same unrelated participants from the Human Connectome Project (HCP; [62]; *n* = 33). We first develop a simple regression-based model to map regional MEG connectivity to regional fMRI connectivity using group-average data. We then investigate how regionally heterogeneous the correspondence between the two is, and how different rhythms contribute to this regional heterogeneity. Finally, we conduct extensive sensitivity testing to demonstrate that the results are robust to multiple methodological choices.

### Relating haemodynamic and electromagnetic connectivity

**Fig 1:**
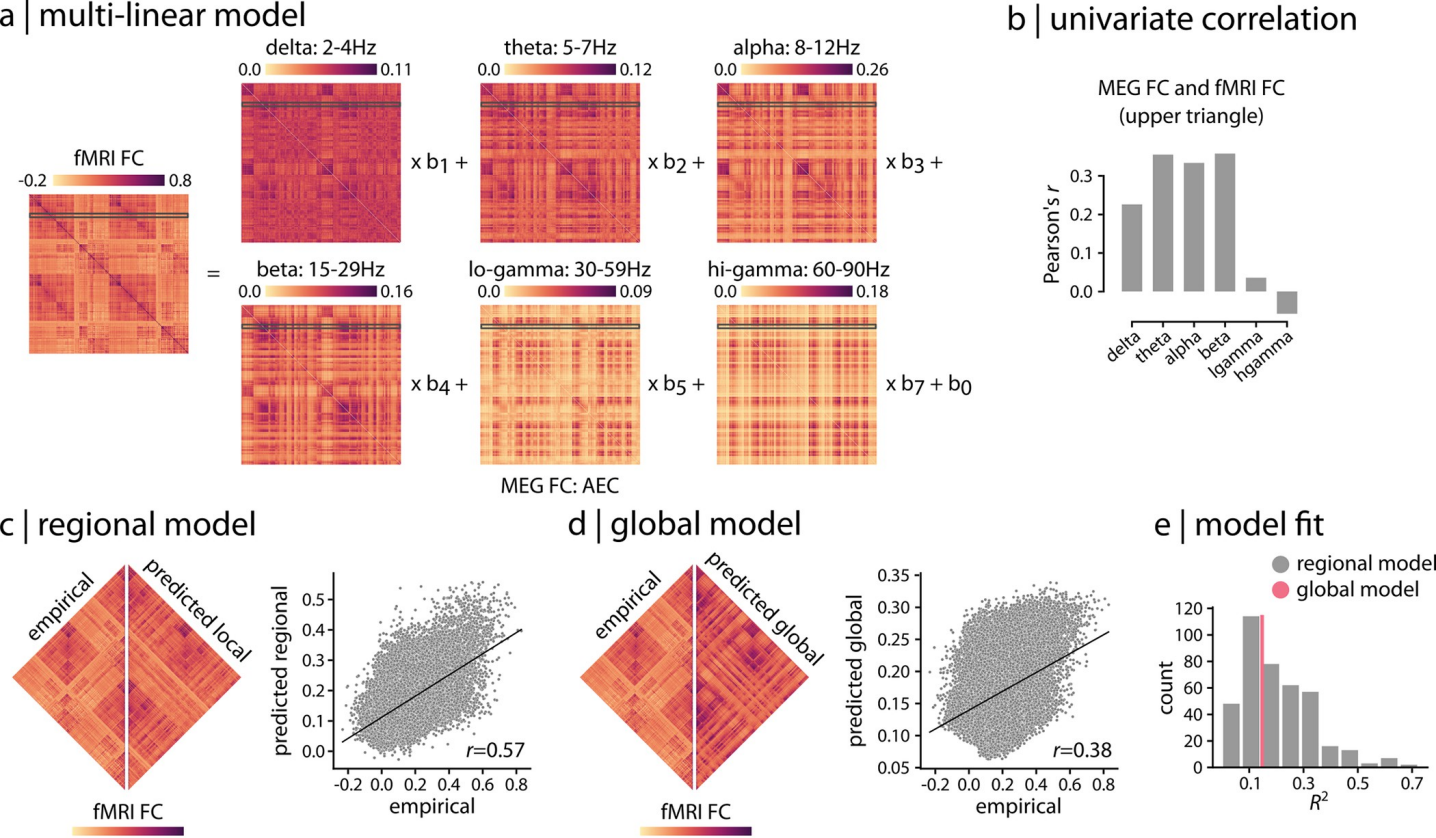
Relating haemodynamic and electromagnetic connectivity. (a) A multilinear regression model was applied to predict resting state fMRI connectivity patterns from band-limited MEG FC (AEC; [60]). The model is specified for each brain region separately, attempting to predict a region’s haemodynamic connectivity profile from its electromagnetic connectivity profile. (b) The overall relationship between fMRI and MEG FC is estimated by correlating the upper triangle of fMRI FC (i.e., above diagonal) with the upper triangles of band-limited MEG FC, suggesting moderate relationship between the two across frequency bands. (c) Regional multilinear model shown in panel (a) is used to predict fMRI FC from band-limited MEG FC for each brain region (i.e., row) separately. The empirical and predicted fMRI FC are depicted side by side for the regional model. The whole-brain edge-wise relationship between the empirical and predicted values is shown in the scatter plot. Each gray dot represents an edge (pairwise functional connection) from the upper triangles of empirical and predicted fMRI FC matrices. (d) A global multilinear model is used to predict the entire upper triangle of fMRI FC from the upper triangles of the MEG FC matrices. The empirical and predicted fMRI FC are depicted side by side for the global model. The whole-brain edge-wise relationship between the empirical and predicted values is shown in the scatter plot. Each gray dot represents en edge from the upper triangles of empirical and predicted fMRI FC matrices. (e) The distribution of regional model fit quantified by *R*^2^ is shown for regional model (gray histogram plot). The global model fit is also depicted for comparison (pink line). The data and code needed to generate this figure can be found in https://github.com/netneurolab/shafiei_megfmrimapping and https://zenodo.org/record/6728338. AEC, amplitude envelope correlation; EEG, electroencephalography; FC, functional connectivity; fMRI, functional magnetic resonance imaging; MEG, magnetoencephalography.

To relate fMRI and MEG FC patterns, we apply a multilinear regression model [68] (Fig 1). The model is specified for each brain region separately, attempting to predict a region’s haemodynamic connectivity profile from its electromagnetic connectivity profile. The dependent variable is a row of the fMRI FC matrix and the independent variables are the corresponding rows of MEG FC matrices for 6 canonical electrophysiological bands, estimated using amplitude envelope correlation (AEC; [60]) with spatial leakage correction (see “Methods” for more details). For a model fitted for a given node *i*, the observations in the model are the connections of node *i* to the other *j*≠*i* regions (Fig 1A). The model predicts the fMRI FC profile of node *i* (i.e., *i*-th row) from a linear combination of MEG FC profiles of node *i* in the 6 frequency bands (i.e., *i*-th rows of MEG FC matrices). Collectively, the model embodies the idea that multiple rhythms could be superimposed to give rise to regionally heterogeneous haemodynamic connectivity.

Indeed, we find that the relationship between haemodynamic and electromagnetic connectivity is highly heterogeneous. Band-limited MEG connectivity matrices are moderately correlated with fMRI connectivity, ranging from *r* = −0.06 to *r* = 0.36 (Fig 1B; *r* denotes Pearson correlation coefficient). The regional multilinear model fits range from adjusted-*R*^2^ = −0.002 to adjusted-*R*^2^ = 0.72 (*R*^2^ denotes coefficient of determination; hereafter we refer to adjusted-*R*^2^ as *R*^2^), suggesting a close correspondence in some regions and poor correspondence in others (Fig 1C and 1E). Band-specific regional model fits are depicted in S1 Fig, where each band-specific MEG connectivity is separately used as a single predictor in the model. For comparison, a single global model is fitted to the data, predicting the entire upper triangle of the fMRI FC matrix (i.e., all values above the diagonal) from a linear combination of the upper triangles of 6 MEG FC matrices (i.e., all values above the diagonal) (see “Methods” for more detail). The global model, which simultaneously relates whole-brain fMRI FC to the whole-brain MEG FC, yields an *R*^2^ = 0.15 (Fig 1D and 1E). Importantly, the global model clearly obscures the wide range of correspondences, which can be considerably greater or smaller for individual regions.

### Hierarchical organization of cross-modal correspondence

**Fig 2:**
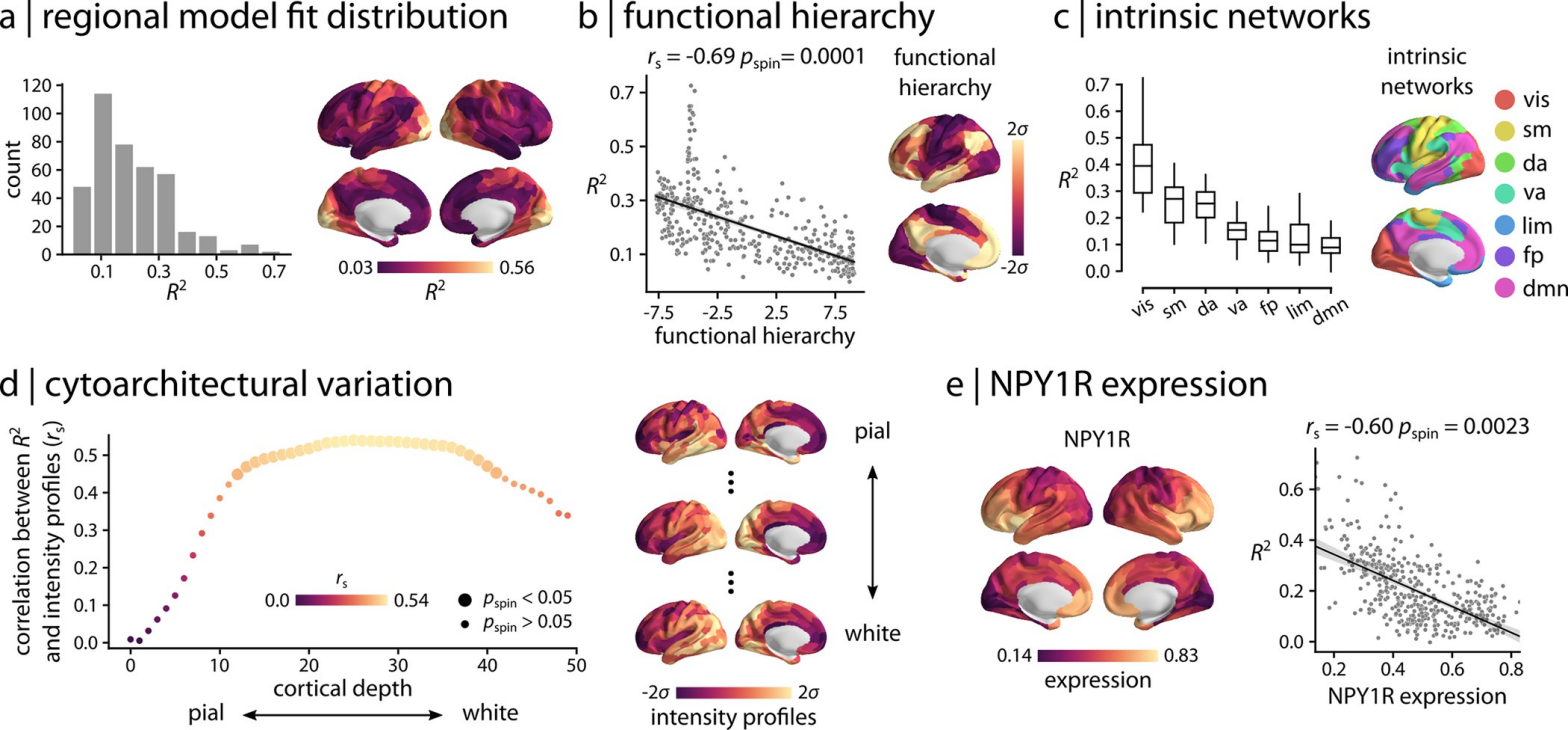
Regional model fit. (a) Spatial organization of fMRI-MEG correspondence is depicted for the regional model fit (95% interval). The cross-modal correspondence of connectivity profiles of brain regions is distributed heterogeneously across the cortex, representing regions with low or high correspondence. Strong cross-modal correspondence is observed in sensory areas, whereas poor correspondence is observed for higher order regions. (b) Spatial organization of the cross-modal correspondence is compared with the functional hierarchical organization of cerebral cortex [63]. The two are significantly anticorrelated, confirming poor fMRI-MEG correspondence in connectivity profile of higher-order, transmodal areas compared to strong correspondence for sensory, unimodal regions. (c) Regions are stratified by their affiliation with macroscale intrinsic networks [2]. The distribution of *R*^2^ is depicted for each network, displaying a systematic gradient of cross-modal correspondence with the highest correspondence in the visual network and lowest correspondence in the default mode network. (d) The model fit is related to the cytoarchitectural variation of the cortex, estimated from the cell staining intensity profiles at various cortical depths obtained from the BigBrain histological atlas [64,65]. Bigger circles denote statistically significant associations after correction for multiple comparisons by controlling the FDR at 5% alpha [66]. The peak association between cross-modal correspondence and cytoarchitecture is observed approximately at cortical layer IV that has high density of granule cells. Staining intensity profiles are depicted across the cortex for the most pial, the middle, and the white matter surfaces. (e) Microarray gene expression of vasoconstrictive NPY1R was estimated from the AHBA [67]. The MEG-fMRI cross-modal correspondence *R*^2^ map (i.e., regional model fit) is compared with NPY1R gene expression. *r*_s_ denotes Spearman rank correlation. Intrinsic networks: vis = visual; sm = somatomotor; da = dorsal attention; va = ventral attention; lim = limbic; fp = frontoparietal; dmn = default mode. The data and code needed to generate this figure can be found in https://github.com/netneurolab/shafiei_megfmrimapping and https://zenodo.org/record/6728338. AHBA, Allen Human Brain Atlas; FDR, false discovery rate; fMRI, functional magnetic resonance imaging; MEG, magnetoencephalography; NPY1R, Neuropeptide Y Receptor Y1.

We next consider the spatial organization of fMRI-MEG correspondence. Fig 2A shows the spatial distribution of regional *R*^2^ values, representing regions with low or high correspondence. Regions with strong cross-modal correspondence include the visual, somatomotor, and auditory cortex. Regions with low cross-modal correspondence include the posterior cingulate, lateral temporal, and medial prefrontal cortex.

Collectively, the spatial layout of cross-modal correspondence bears a resemblance to the unimodal–transmodal cortical hierarchy observed in large-scale functional and microstructural organization of the cerebral cortex [28]. To assess this hypothesis, we first compared the cross-modal *R*^2^ map with the principal functional hierarchical organization of the cortex, estimated using diffusion map embedding [63,71] (Fig 2B; see “Methods” for more details). The two are significantly anticorrelated (Spearman rank correlation coefficient *r*_s_ = −0.69, *p*_spin_ = 0.0001), suggesting strong cross-modal correspondence in unimodal sensory cortex and poor correspondence in transmodal cortex. We then stratify regions by their affiliation with macroscale intrinsic networks and computed the mean *R*^2^ in each network [2] (Fig 2C). Here, we also observe a systematic gradient of cross-modal correspondence, with the strongest correspondence in the visual network and poorest correspondence in the default mode network.

We relate the cross-modal *R*^2^ map to the cytoarchitectural variation of the cortex (Fig 2D). We use the BigBrain histological atlas to estimate granular cell density at multiple cortical depths [64,65]. Cell-staining intensity profiles were sampled across 50 equivolumetric surfaces from the pial surface to the white matter surface to estimate laminar variation in neuronal density and soma size. Fig 2D shows the correlation between MEG-fMRI correspondence and cell density (*y* axis) at different cortical depths (*x* axis). Interestingly, the model fit is associated with cytoarchitectural variation of the cortex, with the peak association observed approximately at cortical layer IV that has high density of granular cells and separates supra- and infragranular layers [72–74]. Layer IV predominately receives feedforward projections and has high vascular density [75–77]. We further assess the relationship between MEG-fMRI cross-modal correspondence and vascular attributes. We obtain the microarray gene expression of the vasoconstrictive NPY1R (Neuropeptide Y Receptor Y1) from Allen Human Brain Atlas (AHBA; [67]; see “Methods” for more details), given previous reports that the BOLD response is associated with the vasoconstrictive mechanism of Neuropeptide Y (NPY) acting on Y1 receptors [78]. We then compare the cross-modal association map with the expression of NPY1R and identify a significant association between the two (Fig 2E; *r*_s_ = −0.60, *p*_spin_ = 0.0023). This demonstrates that regions with low cross-modal correspondence are enriched for NPY1R, whereas areas with high cross-modal associations have less NPY-dependent vasoconstriction. Altogether, the results suggest that the greater coupling in unimodal cortex may be driven by the underlying cytoarchitecture, reflecting higher density of granular cells and distinct vascularization of cortical layer IV.

We also relate cross-modal *R*^2^ map to the variation of structure–function coupling across the cortex, which has also been shown to follow the unimodal–transmodal hierarchy [68,79–82]. We estimate structure–function coupling as the Spearman rank correlation between regional structural and functional connectivity profiles [80] (S2 Fig; see “Methods” for more details). We then correlate the identified map with the regional model fit, identifying a significant association between the two (S2 Fig; *r*_s_ = 0.40, *p*_spin_ = 0.0025). This is consistent with the notion that both haemodynamic and electromagnetic neural activity are constrained by the anatomical pathways and the underlying structural organization [83–85].

### Heterogeneous contributions of multiple rhythms

**Fig 3:**
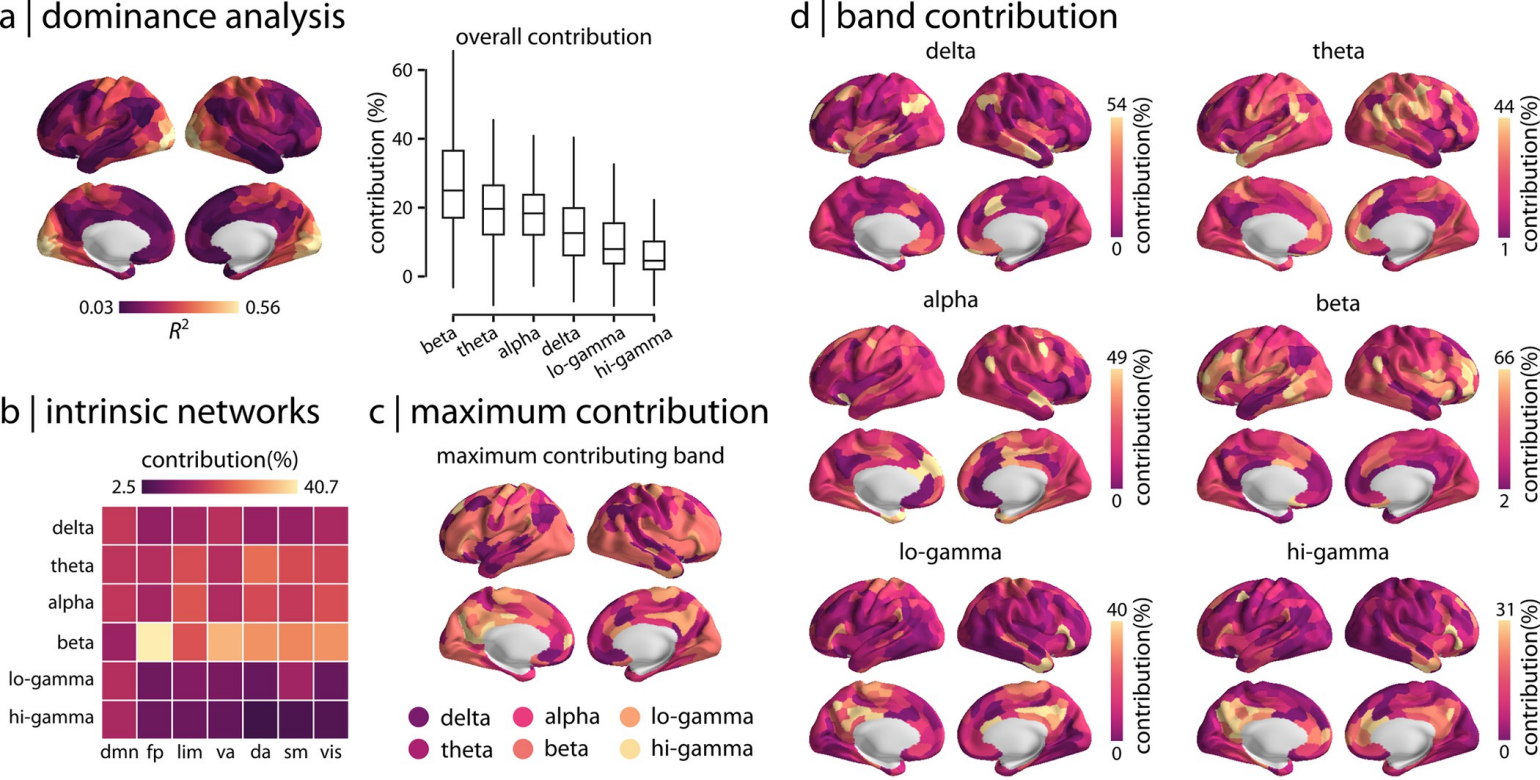
Dominance analysis. Dominance analysis is performed for each regional multilinear model to quantify how MEG connectivity at different rhythms contribute to regional patterns of cross-modal correspondence [69,70]. (a) The overall contribution of each frequency band is depicted for the regional model (box plots). Beta band connectivity, followed by theta and alpha bands, contribute the most to the model fit whereas low and high gamma bands contribute the least. (b) The mean contribution of different rhythms is estimated for the intrinsic networks. Consistent with the overall contributions depicted in panel (a), the greatest contribution is associated with beta band connectivity. (c) The most dominant predictor (frequency band) is depicted for each brain region, confirming overall higher contributions from beta band across the cortex. (d) Frequency band contribution to the regional cross-modal correspondence is shown separately for different rhythms across the cortex (95% intervals). The data and code needed to generate this figure can be found in https://github.com/netneurolab/shafiei_megfmrimapping and https://zenodo.org/record/6728338. MEG, magnetoencephalography.

How do different rhythms contribute to regional patterns of cross-modal correspondence? To address this question and to assess the effects of cross-correlation between MEG connectivity at different frequency bands (S5 Fig), we perform a dominance analysis for every regional multilinear model [69,70]. Specifically, dominance analysis is used to examine the separate effects of each band-limited MEG FC, as well as the effects of all other possible combinations of band-limited MEG FC, on the regional model fit. This technique estimates the relative importance of predictors by constructing all possible combinations of predictors and refitting the multilinear model for each combination. The possible combinations of predictors include sets of single predictors, all possible pairs of predictors, all possible combinations with 3 predictors, and so on. To assess the influence of each band on the model fit, dominance analysis refits the model for each combination and quantifies the relative contribution of each predictor as the increase in variance explained after adding that predictor to the models (i.e., gain in adjusted-*R*^2^). Fig 3A shows the global dominance of each frequency band, where dominance is quantified as “percent relative importance” or “contribution percentage” of each band. Overall, we observe the greatest contributions from MEG connectivity at beta band, followed by theta and alpha bands, and smallest contributions from low and high gamma bands.

Zooming in on individual regions and intrinsic networks, we find that the dominance pattern is also regionally heterogeneous. Namely, the make-up and contribution of specific MEG frequencies to a region’s fMRI connectivity profile varies from region to region. Fig 3B shows the dominance of specific rhythms in each intrinsic network. Fig 3C shows the most dominant predictor for every brain region. We find that beta band contribution is highest in occipital and lateral frontal cortices. Sensorimotor cortex has high contributions from combinations of beta, alpha, and theta bands. Parietal and temporal areas are mostly dominated by delta and theta bands as well as some contribution from alpha band. Medial frontal cortex shows contributions from the alpha band, while low and high gamma bands contribute to posterior cingulate cortex and precuneus. Fig 3D shows the dominance of specific rhythms separately for each region. Overall, we observe that beta connectivity has the highest contribution percentage (95% confidence interval: [2% 66%]), largely contributing to model prediction across the cortex. These findings are consistent with previous reports, demonstrating that haemodynamic connectivity is related to the superposition of band-limited electromagnetic connectivity and that band contributions vary across the cortex [45,47].

Finally, we used analysis of variance (ANOVA) to quantitatively assess the differences in band-specific contributions to the cross-modal correspondence map (S1 Table). Specifically, we assessed the significance and effect size of differences in band-specific contributions for all possible pairs of frequency bands. We identify 2 main findings (for full results, see S1 Table): (1) Overall, the variability of band-specific contributions is significantly larger between groups (i.e., bands) compared to the variability within groups (*F*(5, 2394) = 117.31; *p* < 0.0001). (2) Band-specific contributions are significantly different from each other and are ranked in the same order as depicted in Fig 3A. Specifically, contribution of beta band is significantly larger than contribution of alpha band (difference of the means = 8.65, *t*-value = 9.46, *p*-value < 0.0001, Cohen’s *d* = 0.69) and theta band (difference of the means = 7.56, *t*-value = 8.27, *p*-value < 0.0001, Cohen’s *d* = 0.58). Also, the contribution from the delta band is significantly lower than beta (difference of the means = 12.37, *t*-value = 13.53, *p*-value < 0.0001, Cohen’s *d* = 0.96), alpha (difference of the means = 3.72, *t*-value = 4.07, *p*-value = 0.0007, Cohen’s *d* = 0.29), and theta (difference of the means = 4.81, *t*-value = 5.26, *p*-value < 0.0001, Cohen’s *d* = 0.37). Note that although the difference between alpha and theta band contributions is not significant, both their contributions are significantly lower than beta band and larger than delta band. Moreover, delta band contribution is significantly larger than contribution of lo-gamma (difference of the means = 3.78, *t*-value = 4.14, *p*-value = 0.0005, Cohen’s *d* = 0.29) and lo-gamma contribution is significantly larger than hi-gamma (difference of the means = 3.72, *t*-value = 4.07, *p*-value = 0.0007, Cohen’s *d* = 0.29). Note that the values reported here are the absolute values for difference of the means, *t*-values, *p*-values and Cohen’s *d* (effect size). All *p*-values are corrected for multiple comparisons using Bonferroni correction.

### Sensitivity analysis

**Fig 4:**
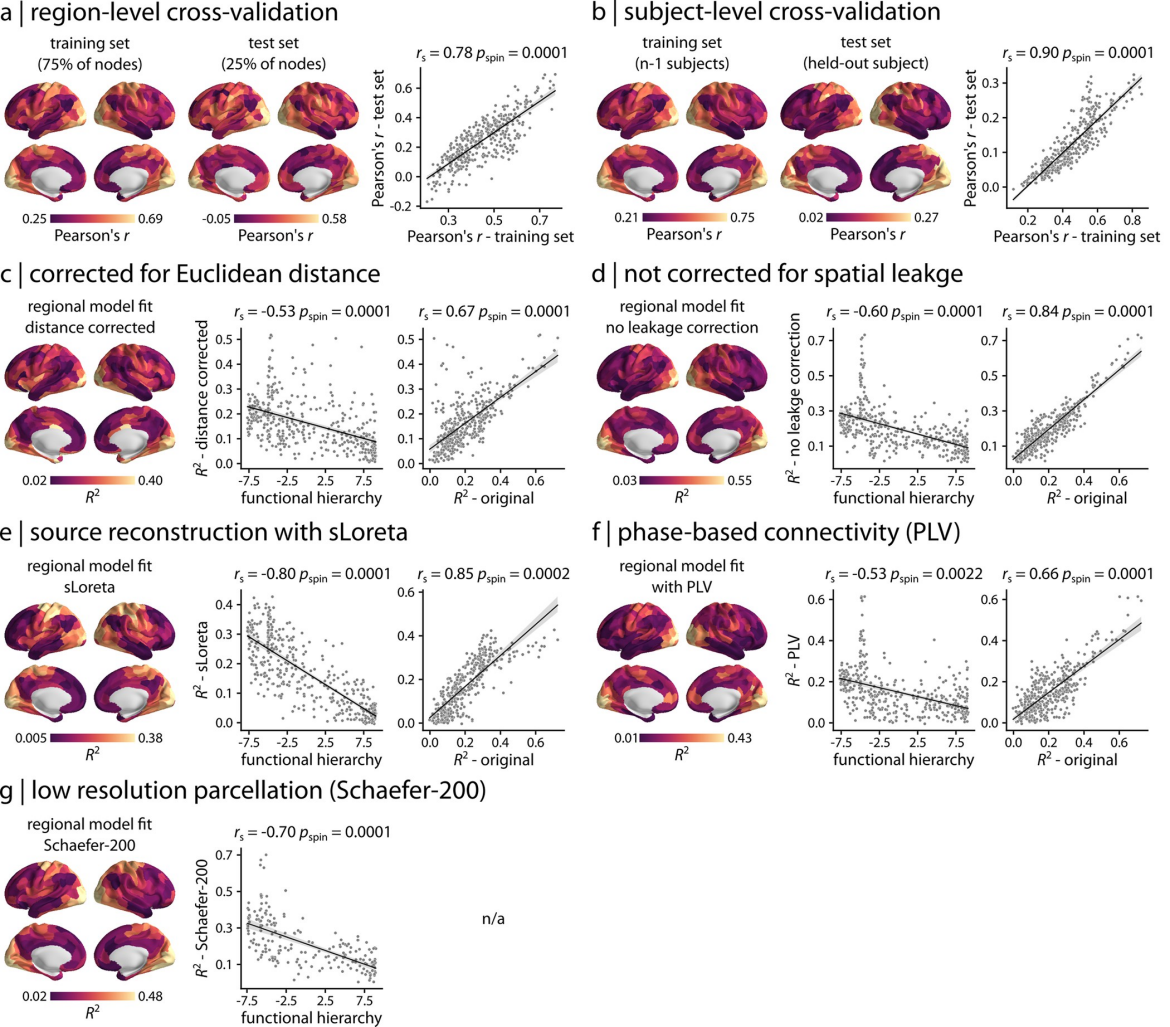
Sensitivity analysis. (a) A regional cross-validation was performed by pseudorandomly splitting the connectivity profile of a given region into train and test sets based on spatial separation (see “Methods” for more details). The multilinear model is then fitted on the train set and is used to predict the connection strength of the test set for each region and each split. The mean regional model performance across splits is depicted for train and test sets, displaying consistent results between the two (scatter plot). The out-of-sample model performance is stronger in the sensory, unimodal areas compared to transmodal areas, consistent with original findings (Fig 2). (b) A subject-level cross-validation was performed using a leave-one-out approach. The regional multilinear model is trained using data from *n*−1 subjects and is tested on the held-out subject for each region separately. The mean regional model performance is shown for train and test sets, displaying consistent results between the two (scatter plot). The out-of-sample model performance is stronger in the sensory, unimodal areas compared to transmodal areas, consistent with original findings (Fig 2). The analysis is also repeated for various processing choices: (c) after regressing out interregional Euclidean distance from connectivity matrices; (d) using MEG connectivity data without spatial leakage correction; (e) using another MEG source reconstruction method (sLoreta; [86]); (f) using a phase-based MEG connectivity measure (PLV; [87,88]); and (g) at a low-resolution parcellation (Schaefer-200 atlas; [89]). The results are consistent across all control analyses, identifying similar cross-modal correspondence maps as the original analysis (Fig 2A). All brain maps are shown at 95% intervals. *r*_s_ denotes Spearman rank correlation. The data and code needed to generate this figure can be found in https://github.com/netneurolab/shafiei_megfmrimapping and https://zenodo.org/record/6728338. MEG, magnetoencephalography; PLV, phase-locking value; sLoreta, standardized low-resolution brain electromagnetic tomography.

Finally, we note that the present report goes through several decision points that have equally justified alternatives. Here, we explore the other possible choices. First, rather than framing the report from an explanatory perspective (focusing on model fit), we instead derive an equivalent set of results using a predictive perspective (focusing on out-of-sample prediction). We perform cross-validation at both the region and subject level (Fig 4A and 4B). For region-level cross-validation, we pseudorandomly split the connectivity profile of a given region into train and test sets based on spatial separation (interregional Euclidean distance), such that 75% of the closest regions to a random region are selected as the train set and the remaining 25% of the regions are selected as test set (399 repetitions; see “Methods” for more details) [90]. We then train the multilinear model using the train set and predict the connection strength of the test set for each region and each split. The mean regional model performance across splits is consistent for train and test sets (Fig 4A; *r* = 0.78, *p*_spin_ = 0.0001). For subject-level cross-validation, we use leave-one-out-cross validation, wherein we train the regional multilinear models using data from *n*−1 subjects and test each one on the held-out subject. The mean regional model performance is consistent for train and test sets (Fig 4B; *r* = 0.90, *p*_spin_ = 0.0001). Altogether, both analyses give similar, highly concordant results with the simpler model fit-based analysis, identifying strong cross-modal correspondence in unimodal sensory regions and poor correspondence in transmodal areas.

To consider the effect of spatial proximity on the findings, we remove the exponential interregional Euclidean distance trend from all connectivity matrices before fitting any model. The results are consistent with and without distance correction (Fig 4C; correlation with functional hierarchy: *r*_s_ = −0.53, *p*_spin_ = 0.0001; correlation with original *R*^2^: *r*_s_ = 0.67, *p*_spin_ = 0.0001). We also obtain consistent findings when we repeat the analysis without accounting for spatial leakage effect in estimating MEG connectivity with AEC (Fig 4D; correlation with functional hierarchy: *r*_s_ = −0.60, *p*_spin_ = 0.0001; correlation with original *R*^2^: *r*_s_ = 0.84, *p*_spin_ = 0.0001). Next, we use another source reconstruction method (standardized low-resolution brain electromagnetic tomography (sLoreta); [86]) instead of linearly constrained minimum variance (LCMV) beamformers, as previous reports suggest that sLoreta improves source localization accuracy [91,92]. We then estimate MEG connectivity with AEC and repeat the multilinear model analysis, identifying similar results as before (Fig 4E; correlation with functional hierarchy: *r*_s_ = −0.80, *p*_spin_ = 0.0001; correlation with original *R*^2^: *r*_s_ = 0.85, *p*_spin_ = 0.0002). Next, we compute MEG connectivity using an alternative, phase-based connectivity measure (phase-locking value (PLV); [87,88]), rather than the AEC. The 2 FC measures yield similar cross-modal correspondence maps (Fig 4F; correlation with functional hierarchy: *r*_s_ = −0.53, *p*_spin_ = 0.0022; correlation with original *R*^2^: *r*_s_ = 0.66, *p*_spin_ = 0.0001). We also repeat the analysis using a low-resolution parcellation (Schaefer-200 atlas; [89]) to ensure that the findings are independent from the choice of parcellation. As before, the results demonstrate similar cross-modal correspondence map (Fig 4G; correlation with functional hierarchy: *r*_s_ = −0.70, *p*_spin_ = 0.0001). To assess the extent to which the results are influenced by MEG source localization error, we compare the cross-modal correspondence pattern to peak localization error estimated using cross-talk function (CTF) [91–95]. No significant association is observed between *R*^2^ pattern and CTF for LCMV (S3A Fig; *r*_s_ = −0.14, *p*_spin_ = 0.6) and sLoreta (S3B Fig; *r*_s_ = −0.04, *p*_spin_ = 0.9) source reconstruction solutions. Finally, to confirm that the cross-modal correspondence pattern is independent from signal-to-noise ratio (SNR), we compare the regional model fit with the SNR map of the reconstructed sources, identifying no significant association between the two (S4 Fig; *r*_s_ = 0.32, *p*_spin_ = 0.25) (see “Methods” for more details).

## Discussion

In the present report, we map electromagnetic functional networks to haemodynamic functional networks in the human brain. We find 2 principal results. First, the relationship between the 2 modalities is regionally heterogeneous but systematic, reflecting the unimodal–transmodal cortical hierarchy and cytoarchitectural variation. Second, haemodynamic connectivity cannot be explained by electromagnetic connectivity in a single band, but rather reflects mixing and superposition of multiple rhythms.

The fact that the association between the 2 modalities follows a gradient from unimodal to transmodal cortex resonates with emerging work on cortical hierarchies [28,63,96]. Indeed, similar spatial variations are observed for multiple microarchitectural features, such as gene expression [90,97,98], T1w/T2w ratio [99], laminar differentiation [74], and neurotransmitter receptor profiles [100–102]. Collectively, these studies point to a natural axis of cortical organization that encompasses variations in both structure and function across micro-, meso-, and macroscopic spatial scales.

Interestingly, we find the closest correspondence between fMRI and MEG FC in unimodal cortex (including the visual and somatomotor networks) and the poorest correspondence in transmodal cortex (default mode, limbic, frontoparietal, and ventral attention networks). In other words, the functional architectures of the 2 modalities are consistent early in the cortical hierarchy, presumably reflecting activity related to instantaneous changes in the external environment. Conversely, as we move up the hierarchy, there is a gradual separation between the 2 architectures, suggesting that they are differently modulated by endogenous inputs and contextual information. How the 2 types of FC are related to ongoing task demand is an exciting question for future research.

Why is there systematic divergence between the 2 modalities? Our findings suggest that topographic variation in MEG-fMRI coupling is due to variation in cytoarchitecture and neurovascular coupling. First, we observe greater MEG-fMRI coupling in regions with prominent granular layer IV. This result may reflect variation of microvascular density at different cortical layers [59,77,103]. Namely, cortical layer IV is the most vascularized, and this is particularly prominent in primary sensory areas [77]. The BOLD response mainly reflects local field potentials arising from synaptic currents of feedforward input signals to cortical layer IV [75,76]; as a result, the BOLD response is more sensitive to cortical layer IV with high vascular density [104]. Therefore, electromagnetic neuronal activity originating from layer IV should be accompanied by a faster and more prominent BOLD response. This is consistent with our finding that brain regions with more prominent granular layer IV (i.e., unimodal cortex) have greater correspondence between electromagnetic and haemodynamic functional architectures. In other words, heterogeneous cortical patterning of MEG-fMRI coupling may reflect heterogeneous patterning of underlying neurovascular coupling.

Second, we observe prominent anticorrelations between vasoconstrictive NPY1R-expressing neurons and MEG-fMRI coupling. Multiple studies of vasodilator and vasoconstrictor mechanisms involved in neural signaling have demonstrated links between microvasculature and the BOLD signal [78,103]. For example, an optogenetic and 2-photon mouse imaging study found that task-related negative BOLD signal is mainly associated with vasoconstrictive mechanism of NPY acting on Y1 receptors, suggesting that neurovascular coupling is cell specific [78]. Interestingly, by comparing the cortical expression of NPY1R in the human brain with MEG-fMRI correspondence pattern identified here, we find that regions with low cross-modal correspondence are enriched for NPY1R, whereas areas with high cross-modal associations have less NPY-dependent vasoconstriction. Collectively, these results suggest that MEG-fMRI correspondence is at least partly due to regional variation in cytoarchitecture and neurovascular coupling.

More generally, numerous studies have investigated the laminar origin of cortical rhythms. For example, animal electrophysiological recordings demonstrated that visual and frontal cortex gamma activity can be localized to superficial cortical layers (supragranular layers I to III and granular layer IV), whereas alpha and beta activity are localized to deep infragranular layers (layers V to VI) [52–56,58]. Similar findings have been reported in humans using EEG and laminar-resolved BOLD recordings, demonstrating that gamma and beta band EEG power are associated with superficial and deep layer BOLD response, respectively, whereas alpha band EEG power is associated with BOLD response in both superficial and deep layers [57]. Laminar specificity of cortical rhythms is increasingly emphasized in contemporary accounts of predictive processing [105]. In the predictive coding framework, transmodal regions generate predictive signals that modulate the activity of sensory unimodal regions depending on context [106]. These top-down signals are relatively slow, as they evolve with the context of exogenous (stimulation) inputs. The consequence on unimodal areas is a boost of their encoding gain, reflected in stronger, faster activity that tracks incoming stimuli. They in turn generate error signals that are slower and reflect the discrepancy between the predictions received and the actual external input. These slower error signals are then registered by higher-order transmodal regions. Specific cortical layers and rhythms contribute to this predictive coding [105]. For example, an unfamiliar, unpredicted stimulus is associated with increased gamma power that is fed forward up the cortical hierarchy (i.e., bottom-up from sensory to association cortices) through the superficial layers to transfer the prediction errors. This in turn results in low top-down, feedback predictions through deep cortical layers via alpha and beta rhythms. Conversely, predicted stimuli are associated with stronger feedback alpha and beta rhythms via deep layers, inhibiting the gamma activity for expected exogenous inputs [105]. This hierarchical predictive processing framework is also thought to underlie conscious perception by top-down transfer of perceptual predictions via alpha and beta rhythms through deep layers and bottom-up transfer of prediction errors via gamma rhythm through superficial layers, minimizing predictions errors [105,107,108]. Our results, linking cytoarchitecture with rhythm-specific connectivity, may help to further refine and develop this emerging framework.

Altogether, our findings suggest that the systemic divergence between MEG and fMRI connectivity patterns may reflect variations in cortical cytoarchitecture and vascular density of cortical layers. However, note that due to the low spatial resolution of fMRI and MEG data, haemodynamic and electromagnetic connectivity is not resolved at the level of cortical layers. Rather, comparisons with cytoarchitecture are made via proxy datasets, such as the BigBrain histological atlas [64] and the AHBA [67]. Future work is required to assess the laminar specificity of the cross-modal association in a more direct and comprehensive manner [109–112].

Throughout the present report, we find that fMRI networks are best explained as arising from the superposition of multiple band-limited MEG networks. Although previous work has focused on directly correlating fMRI with MEG/EEG networks in specific bands, we show that synchronized oscillations in multiple bands could potentially combine to give rise to the well-studied fMRI functional networks. Indeed, and as expected, the correlation between any individual band-specific MEG network and fMRI is substantially smaller than the multilinear model that takes into account all bands simultaneously. Previous work on cross-frequency interactions [113] and on multilayer MEG network organization [114] has sought to characterize the participation of individual brain regions within and between multiple frequency networks. Our findings build on this literature, showing that the superimposed representation may additionally help to unlock the link between MEG and fMRI networks.

It is noteworthy that the greatest contributions to the link between the 2 modalities came from beta band connectivity. Multiple authors have reported that—since it captures slow haemodynamic coactivation—fMRI network connectivity would be mainly driven by slower rhythms [6,20,35,42,61,113]. Our findings demonstrate that although all frequency bands contribute to the emergence of fMRI networks, the greatest contributions come from beta band connectivity, followed by theta and alpha connectivity.

The present results raise 2 important questions for future work. First, how does structural connectivity shape fMRI and MEG functional networks [43,81,83]? We find that cross-modal correspondence between MEG and fMRI functional networks is associated with structure–function coupling measured from MRI functional and structural connectivity networks, suggesting that the cross-modal map may be constrained by structural connectivity. Previous reports demonstrate that unimodal, sensory regions have lower neural flexibility compared to transmodal, association areas and are more stable during development and evolution [24,115,116]. This suggests that the underlying anatomical network constrains neural activity and functional flexibility in a nonuniform manner across the cortex, resulting in higher degrees of freedom in structure–function coupling in regions related to highly flexible cognitive processes. However, given that MEG and fMRI capture distinct neurophysiological mechanisms, it is possible that haemodynamic and electromagnetic architectures have a different relationship with structural connectivity, and this could potentially explain why they systematically diverge through the cortical hierarchy [68,79–82]. Second, the present results show how the 2 modalities are related in a task-free resting state, but what is the relationship between fMRI and MEG connectivity during cognitive tasks [117]? Given that the 2 modalities become less correlated in transmodal cortex in the resting state, the relationship between them during task may depend on demand and cognitive functions required to complete the task.

Finally, the present results should be interpreted in light of several methodological considerations. First, although we conduct extensive sensitivity testing, including multiple ways of defining FC, there exist many more ways in the literature to estimate both fMRI and MEG connectivity [118,119]. Second, to ensure that the analyses were performed in the same participants using both resting state fMRI and MEG and that the participants have no familial relationships, we utilized a reduced version of the HCP sample. Third, in order to directly compare the contributions of multiple frequency bands, all were entered into the same model. As a result, however, the observations in the linear models (network edges) are not independent, violating a basic assumption of these statistical models. For this reason, we only use model fits and dominance values to compare the correspondence of fMRI and MEG across a set of nodes, each of which is estimated under the same conditions. Finally, to ensure that the findings are independent from sensitivity of MEG to neural activity from different regions, we compared the cross-modal correspondence map with MEG SNR and source localization error, where no significant associations were identified. However, MEG is still susceptible to such artifacts given that regions with lower SNR (e.g., Sylvian fissure) are the ones where source reconstruction solutions have higher source localization errors [120,121].

Despite complementary strengths to image spatiotemporal brain dynamics, the links between MEG and fMRI are not fully understood and the 2 fields have diverged. The present report bridges the 2 disciplines by comprehensively mapping haemodynamic and electromagnetic network architectures. By considering the contributions of the canonical frequency bands simultaneously, we show that the superposition and mixing of MEG neurophysiological rhythms manifests as highly structured patterns of fMRI FC. Systematic convergence and divergence among the 2 modalities in different brain regions opens fundamentally new questions about the relationship between cortical hierarchies and multimodal functional networks.

## Methods

### Dataset: Human Connectome Project (HCP)

Resting state MEG data of a set of healthy young adults (*n* = 33; age range 22 to 35 years) with no familial relationships were obtained from HCP (S900 release; [62]). The data include resting state scans of about 6 minutes long (sampling rate = 2,034.5 Hz; anti-aliasing filter low-pass filter at 400 Hz) and noise recordings for all participants. MEG anatomical data and 3T structural magnetic resonance imaging (MRI) data of all participants were also obtained for MEG preprocessing. Finally, we obtained functional MRI data of the same *n* = 33 individuals from HCP dataset. All 4 resting state fMRI scans (2 scans with R/L and L/R phase encoding directions on day 1 and day 2, each about 15 minutes long; *TR* = 720 ms) were available for all participants.

### HCP data processing

#### Resting state magnetoencephalography (MEG)

Resting state MEG data were analyzed using Brainstorm software, which is documented and freely available for download online under the GNU general public license ([122]; http://neuroimage.usc.edu/brainstorm). The MEG recordings were registered to the structural MRI scan of each individual using the anatomical transformation matrix provided by HCP for coregistration, following the procedure described in Brainstorm’s online tutorials for the HCP dataset (https://neuroimage.usc.edu/brainstorm/Tutorials/HCP-MEG). The preprocessing was performed by applying notch filters at 60, 120, 180, 240, and 300 Hz, and was followed by a high-pass filter at 0.3 Hz to remove slow-wave and DC-offset artifacts. Bad channels were marked based on the information obtained through the data management platform of HCP for MEG data (ConnectomeDB; https://db.humanconnectome.org/). The artifacts (including heartbeats, eye blinks, saccades, muscle movements, and noisy segments) were then removed from the recordings using automatic procedures as proposed by Brainstorm. More specifically, electrocardiogram (ECG) and electrooculogram (EOG) recordings were used to detect heartbeats and blinks, respectively. We then used Signal–Space Projections (SSPs) to automatically remove the detected artifacts. We also used SSP to remove saccades and muscle activity as low-frequency (1 to 7 Hz) and high-frequency (40 to 240 Hz) components, respectively.

The preprocessed sensor-level data were then used to obtain a source estimation on HCP’s fsLR4k cortex surface for each participant. Head models were computed using overlapping spheres, and the data and noise covariance matrices were estimated from the resting state MEG and noise recordings. LCMV beamformers method from Brainstorm was then used to obtain the source activity for each participant. We performed data covariance regularization and normalized the estimated source variance by the noise covariance matrix to reduce the effect of variable source depth. The L2 matrix norm (i.e., regularization parameter) of data covariance matrix is usually defined as the largest eigenvalue of its eigenspectrum. However, the eigenspectrum of MEG data covariance can be ill-conditioned, such that the eigenvalues may span many decades where larger eigenvalues are overestimated and smaller eigenvalues are underestimated. In other words, the L2 norm of the data covariance matrix can be many times larger than the majority of eigenvalues, making it difficult to select a conventional regularization parameter. Following guidelines from Brainstorm [122], we used the “median eigenvalue” method to regularize the data covariance matrix, where the eigenvalues smaller than the median eigenvalue are replaced with the median eigenvalue itself (i.e., flattening the tail of eigenvalues spectrum to the median). The covariance matrix is then reconstructed using the modified eigenspectrum. This helps to avoid the instability of data covariance inversion caused by the smallest eigenvalues and regularizes the data covariance matrix. Source orientations were constrained to be normal to the cortical surface at each of the 8,000 vertex locations on the fsLR4k surface. Source-level time-series were then parcellated into 400 regions using the Schaefer-400 atlas [89], such that a given parcel’s time series was estimated as the first principal component of its constituting sources’ time series.

Parcellated time-series were then used to estimate FC with an amplitude-based connectivity measure from Brainstorm (AEC; [60]). An orthogonalization process was applied to correct for the spatial leakage effect by removing all shared zero-lag signals [123]. AEC FC were derived for each participant at 6 canonical electrophysiological bands (i.e., delta (*δ*: 2 to 4 Hz), theta (*θ*: 5 to 7 Hz), alpha (*α*: 8 to 12 Hz), beta (*β*: 15 to 29 Hz), low gamma (lo-*γ*: 30 to 59 Hz), and high gamma (hi-*γ*: 60 to 90Hz)). Group-average MEG FC matrices were constructed as the mean FC across all individuals for each frequency band. For comparison, band-limited group-average AEC matrices were also estimated without correcting for spatial leakage effect.

We also processed the MEG data using additional methodological choices. First, the LCMV source reconstructed and parcellated time-series were used to estimate FC with an alternative, phase-based connectivity measure (PLV; [87,88]) for each frequency band. Second, another source reconstruction method (sLoreta; [86]) was used instead of LCMV beamformers to obtain source-level time-series, given that previous reports suggest that sLoreta improves source localization accuracy [91,92]. Source-level time-series, obtained by sLoreta, were then parcellated into 400 regions and were used to estimate AEC matrices with spatial leakage correction for the 6 frequency bands. Third, to ensure that the findings are independent from choice of parcellation, a low-resolution atlas (Schaefer-200; [89]) was used to parcellate the original LCMV source-level time-series to 200 cortical regions and obtain spatial leakage corrected AEC connectivity matrices. Finally, we estimated MEG source localization errors for LCMV and sLoreta source reconstruction solutions using CTFs [91–95,121]. CTF of a given source *i* is a measure of how activity from all other sources contributes to the activity estimated for the *i*-th source. Following guidelines from Brainstorm [122] and MNE-Python software packages [124], we used CTF to calculate peak localization error of a given source *i* as the Euclidean distance between the peak location estimated for source *i* and the true source location *i* on the surface model [92,95]. Source-level CTF was then parcellated using the Schaefer-400 atlas. We also estimated source-level SNR for LCMV source reconstruction solution as follows [120,125]:

$$\mathrm{SNR}=10\log10(\frac{a^{2}}{N}\overset{k=1}{\underset{N}{\sum }}\frac{b_{k}^{2}}{s_{k}^{2}})$$ (1)

where *a* is the source amplitude (i.e., typical strength of a dipole, which is 10 nAm; [126]), *N* is the number of sensors, *b*_*k*_ is the signal at sensor *k* estimated by the forward model for a source with unit amplitude, and $s_{k}^{2}$ is the noise variance at sensor *k*. SNR was first calculated at the source level and was then parcellated using the Schaefer-400 atlas.

#### Resting state functional MRI

The functional MRI data were preprocessed using HCP minimal preprocessing pipelines [62,127]. Detailed information regarding data acquisition and preprocessing is available elsewhere [62,127]. Briefly, all 3T functional MRI time-series (voxel resolution of 2 mm isotropic) were corrected for gradient nonlinearity, head motion using a rigid body transformation, and geometric distortions using scan pairs with opposite phase encoding directions (R/L, L/R) [128]. Further preprocessing steps include coregistration of the corrected images to the T1w structural MR images, brain extraction, normalization of whole brain intensity, high-pass filtering (>2,000s FWHM; to correct for scanner drifts), and removing additional noise using the ICA-FIX process [128,129]. The preprocessed time-series were then parcellated into 400 cortical areas using Schaefer-400 parcellation [89]. The parcellated time-series were used to construct FC matrices as Pearson correlation coefficients between pairs of regional time-series for each of the 4 scans and each participant. A group-average FC matrix was constructed as the mean FC across all individuals and scans.

#### Diffusion weighted imaging (DWI)

Diffusion weighted imaging (DWI) data were obtained for the same individuals from the HCP dataset. MRtrix3 package [130] (https://www.mrtrix.org/) was used to preprocess the DWI data as described elsewhere [24]. In brief, multishell multitissue constrained spherical deconvolution algorithm from MRtrix was applied to generate fiber orientation distributions [131,132]. Probabilistic streamline tractography based on the generated fiber orientation distributions was used to reconstruct white matter edges [133]. The tract weights were optimized by estimating an appropriate cross-section multiplier for each streamline following the procedure proposed by Smith and colleagues [134]. Structural connectivity matrices were then reconstructed for each participant using the Schaefer-400 atlas [89]. Finally, a binary group-level structural connectivity matrix was constructed using a consensus approach that preserves the edge length distribution in individual participants [135,136]. The binary consensus structural connectivity matrix was weighted by the average structural connectivity across individuals to obtain a weighted structural connectivity matrix.

### BigBrain histological data

To characterize the cytoarchitectural variation across the cortex, cell-staining intensity profile data were obtained from the BigBrain atlas [64,65]. The BigBrain is a high-resolution (20 *μm*) histological atlas of a postmortem human brain and includes cell-staining intensities that are sampled at each vertex across 50 equivolumetric surfaces from the pial to the white matter surface using the Merker staining technique [64,137]. The staining intensity profile data represent neuronal density and soma size at varying cortical depths, capturing the regional differentiation of cytoarchitecture [64,65,72,74,138]. Intensity profiles at various cortical depths can be used to approximately identify boundaries of cortical layers that separate supragranular (cortical layers I to III), granular (cortical layer IV), and infragranular (cortical layers V to VI) layers [65,74,138]. The data were obtained on *fsaverage* surface (164k vertices) from the BigBrainWarp toolbox [65] and were parcellated into 400 cortical regions using the Schaefer-400 atlas [89].

The cross-modal correspondence map, estimated as adjusted-*R*^2^ (see “Multilinear model” for more details), was then compared with the parcellated cell-staining intensity data. Specifically, the regional model fit was correlated with cell-staining profiles at each cortical depth using Spearman rank correlation (*r*_*s*_). 10,000 spatial-autocorrelation preserving nulls were used to construct a null distribution of correlation at each cortical depth (see “Null model” for more details on spatial-autocorrelation preserving nulls). Significance of the associations were estimated by comparing the empirical Spearman rank correlation with the distribution of null correlations at each cortical depth, identifying the number of null correlations that were equal to or greater than the empirical correlation (two-tailed test). Finally, Benjamini–Hochberg procedure [66] was used to correct for multiple comparisons by controlling the false discovery rate (FDR) at 5% across all 50 comparisons.

### Allen Human Brain Atlas (AHBA)

Regional microarray expression data were obtained from 6 postmortem brains (1 female, ages 24.0 to 57.0, 42.50 ± 13.38) provided by the AHBA (https://human.brain-map.org; [67]). Data were processed with the abagen toolbox (version 0.1.3-doc; https://github.com/rmarkello/abagen; [139]) using the Schaefer-400 volumetric atlas in MNI space [89].

First, microarray probes were reannotated using data provided by [140]; probes not matched to a valid Entrez ID were discarded. Next, probes were filtered based on their expression intensity relative to background noise [141], such that probes with intensity less than the background in ≥50.00% of samples across donors were discarded. When multiple probes indexed the expression of the same gene, we selected and used the probe with the most consistent pattern of regional variation across donors (i.e., differential stability; [142]), calculated with

$$\Delta_{S}(p)=\frac{1}{\left(\begin{array}{l} N\\ 2 \end{array}\right)}\sum_{i=1}^{N-1}‍\sum_{j=i+1}^{N}‍\rho [B_{i}(p),B_{j}(p)]$$

where *ρ* is Spearman’s rank correlation of the expression of a single probe, *p*, across regions in 2 donors *B*_*i*_ and *B*_*j*_, and *N* is the total number of donors. Here, regions correspond to the structural designations provided in the ontology from the AHBA.

The MNI coordinates of tissue samples were updated to those generated via nonlinear registration using the Advanced Normalization Tools (ANTs; https://github.com/chrisfilo/alleninf). To increase spatial coverage, tissue samples were mirrored bilaterally across the left and right hemispheres [143]. Samples were assigned to brain regions in the provided atlas if their MNI coordinates were within 2 mm of a given parcel. If a brain region was not assigned a tissue sample based on the above procedure, every voxel in the region was mapped to the nearest tissue sample from the donor in order to generate a dense, interpolated expression map. The average of these expression values was taken across all voxels in the region, weighted by the distance between each voxel and the sample mapped to it, in order to obtain an estimate of the parcellated expression values for the missing region. All tissue samples not assigned to a brain region in the provided atlas were discarded.

Intersubject variation was addressed by normalizing tissue sample expression values across genes using a robust sigmoid function [144]:

$$x_{norm}=\frac{1}{1+exp(-\frac{\left(x-\langle x\rangle \right)}{IQR_{x}})}$$

where 〈*x*〉 is the median and IQR_*x*_ is the normalized interquartile range of the expression of a single tissue sample across genes. Normalized expression values were then rescaled to the unit interval:

$$x_{scaled}=\frac{x_{norm}-min(x_{norm})}{max(x_{norm})-min(x_{norm})}$$

Gene expression values were then normalized across tissue samples using an identical procedure. Samples assigned to the same brain region were averaged separately for each donor and then across donors, yielding a regional expression matrix of 15,633 genes. Expression of NPY1R was extracted from the regional expression matrix and was related to the cross-modal correspondence map, estimated as adjusted-*R*^2^ (see “Multilinear model” for more details), using 10,000 spatial-autocorrelation preserving nulls (see “Null models” for more details).

### Multilinear model

#### Regional model

A multiple linear regression model was used to assess regional associations between haemodynamic (fMRI) and electromagnetic (MEG) FC (Fig 1; [68]). A separate multilinear model is applied for each brain region from the parcellated data, predicting the region’s fMRI FC profile from its band-limited MEG FC. The dependent variable is a row of the fMRI connectivity matrix and the independent variables (predictors) are the corresponding rows of MEG connectivity for the 6 canonical electrophysiological bands. The linear regression model for each brain region *i* is constructed as follows:

$$\begin{array}{ll} FC_{i}= & b_{1}\times FC(\delta {)}_{i}+b_{2}\times FC(\theta {)}_{i}+\\ & b_{3}\times FC(\alpha {)}_{i}+b_{4}\times FC(\beta {)}_{i}+\\ & b_{5}\times FC(lo,\gamma {)}_{i}+b_{6}\times FC(hi,\gamma {)}_{i}+b_{0} \end{array}$$ (2)

where the dependant variable *FC*_*i*_ is the set of fMRI connections of node *i* to the other *j*≠*i* regions and the predictors are sets of MEG connections of node *i* to the other *j*≠*i* regions for the 6 frequency bands ($FC(\delta {)}_{i},\;FC(\theta {)}_{i},\;FC(\alpha {)}_{i},\;FC(\beta {)}_{i},\;FC(lo,\gamma {)}_{i}$, and *FC*(hi, *γ*)_*i*_). The regression coefficients *b*_1_,…,*b*_6_ and the intercept *b*_0_ are then optimized to yield maximum correlation between empirical and predicted fMRI connectivity for each brain region. Goodness of fit for each regional model is quantified using adjusted-*R*^2^ (coefficient of determination).

#### Global model

For comparison with the regional model, a single global model was fitted to the data, predicting the whole-brain fMRI FC from the whole-brain band-limited MEG FC (Fig 1D). Specifically, rather than applying a multilinear model for each region (i.e., each row) separately, we fit a single multilinear model using the upper triangle of band-limited MEG connectivity (i.e., all values above the diagonal of MEG connectivity matrices) as predictors and predict the upper triangle of fMRI connectivity. The equation below describes the multilinear global model:

$$\begin{array}{ll} FC_{UT}= & b_{1}\times FC(\delta {)}_{UT}+b_{2}\times FC(\theta {)}_{UT}+\\ & b_{3}\times FC(\alpha {)}_{UT}+b_{4}\times FC(\beta {)}_{UT}+\\ & b_{5}\times FC(lo,\gamma {)}_{UT}+b_{6}\times FC(hi,\gamma {)}_{UT}+b_{0} \end{array}$$ (3)

where the dependent variable *FC*_*UT*_ is the vectorized upper triangle of fMRI FC (i.e., above diagonal values) and the predictors are the vectorized upper triangles of MEG FC for the 6 frequency bands. The regression coefficients *b*_1_,…,*b*_6_ and the intercept *b*_0_ are then optimized to yield maximum correlation between empirical and predicted fMRI connectivity. Similar to the regional model, the goodness of fit for the global model is quantified using adjusted-*R*^2^ (coefficient of determination).

#### Region-level cross-validation

Region-level cross-validation was performed to assess out-of-sample model performance. Given the spatial autocorrelation inherent to the data, random splits of brain regions into train and test sets may result in out-of-sample correlations that are inflated due to spatial proximity [145]. To take this into account, we used a distance-dependent cross-validation approach where we pseudorandomly split the connectivity profile of a given region (e.g., node *i*) into train and test sets based on spatial separation [90]. We used interregional Euclidean distance to select 75% of the closest regions to a randomly selected source region as the train set and the remaining 25% of the regions as test set. The random source region can be any of the 399 regions connected to node *i*; hence, the connectivity profile of node *i* is split into 399 unique train and test sets. We then train the multilinear model using the train set and predict FC of the test set for each region and each split. Finally, the model performance is quantified using Pearson correlation coefficient between empirical and predicted values. The cross-validated regional model performance is then estimated as the mean correlation coefficient between empirical and predicted values across splits for each brain region.

#### Subject-level cross-validation

Leave-one-out cross-validation was performed to assess model performance on held-out subjects. Briefly, the regional multilinear model is trained using the group-average data from *n*−1 subjects. The trained model is then used to predict fMRI connectivity profile of each region on the held-out subject (test set). The model performance is quantified as the Pearson correlation coefficient between empirical and predicted connectivity of each region. The analysis is repeated for all subjects, and the regional model performance is averaged across individuals.

### Diffusion map embedding

Diffusion map embedding was used to identify the principal axis of variation in functional organization of the cortex (diffusion map embedding and alignment package; https://github.com/satra/mapalign) [63,71]. Diffusion map embedding is a nonlinear dimensionality reduction technique that generates a low-dimensional representation of high-dimensional data by projecting it into an embedding space, such that the areas with similar connectivity profiles will be closer in distance in the new common space compared to the areas with dissimilar connectivity profiles [63,71,146]. In brief, following the procedure described by Margulies and colleagues [63], each row of the group-average fMRI FC was thresholded at 90%, such that only the top 10% of functional connections was retained in the matrix. Next, a cosine-similarity matrix was estimated based on the remaining functional connections, where the resulting pairwise cosine distances represent the similarity between the connectivity profiles of cortical regions according to their strongest connections. Finally, the diffusion map embedding was applied to the resulting positive affinity matrix. This identifies the principal axis of variation in FC, along which cortical regions are ordered based on the similarity of their connectivity profiles. The identified functional gradient or hierarchy spans the unimodal–transmodal axis, separating primary sensory-motor cortices from association cortex. The functional gradient map is also available as part of the neuromaps toolbox [147]. The functional gradient was used as a metric of hierarchical organization of the cortex and was compared with the regional model fit (Fig 2).

### Structure–function coupling

Structure–function coupling was estimated following the procedure described by Baum and colleagues [80]. Structural and functional connectivity profiles of each brain region (i.e., each row of the connectivity matrices) were extracted from the weighted group-level structural and functional connectivity matrices. Structure–function coupling of a given region was then estimated as the Spearman rank correlation between nonzero values of that region’s structural and functional connectivity profiles. Finally, the resulting whole-brain structure–function coupling map was compared with the cross-modal correspondence map (i.e., *R*^2^ map from the regional model). Significance of the association between the 2 maps was assessed using 10,000 spatial-autocorrelation preserving nulls (see “Null model” for more details).

### Dominance analysis

Dominance analysis was used to quantify the distinct contributions of resting state MEG connectivity at different frequency bands to the prediction of resting state fMRI connectivity in the multilinear model [69,70] (https://github.com/dominance-analysis/dominance-analysis). Dominance analysis estimates the relative importance of predictors by constructing all possible combinations of predictors and refitting the multilinear model for each combination (a model with *p* predictors will have 2^*p*^−1 models for all possible combinations of predictors). The relative contribution of each predictor is then quantified as increase in variance explained by adding that predictor to the models (i.e., gain in adjusted-*R*^2^). Here, we first constructed a multiple linear regression model for each region with MEG connectivity profile of that region at 6 frequency bands as independent variables (predictors) and fMRI connectivity of the region as the dependent variable to quantify the distinct contribution of each factor using dominance analysis. The relative importance of each factor is estimated as “percent relative importance,” which is a summary measure that quantifies the percent value of the additional contribution of that predictor to all subset models.

### Null model

To make inferences about the topographic correlations between any 2 brain maps, we implement a null model that systematically disrupts the relationship between 2 topographic maps but preserves their spatial autocorrelation [145,148]. We used the Schaefer-400 atlas in the HCP’s fsLR32k grayordinate space [62,89]. The spherical projection of the fsLR32k surface was used to define spatial coordinates for each parcel by selecting the vertex closest to the center-of-mass of each parcel [68,149,150]. The resulting spatial coordinates were used to generate null models by applying randomly sampled rotations and reassigning node values based on the closest resulting parcel (10,000 repetitions). The rotation was applied to one hemisphere and then mirrored to the other hemisphere.

## Supporting information

S1 Table Analysis of variance (ANOVA) for dominance analysis. To quantitatively assess the differences in band-specific contributions to the cross-modal correspondence map, contributions estimated from dominance analysis were compared for all possible pairs of frequency bands using analysis of variance (ANOVA). All reported *p*-values are from two-tailed tests and are corrected for multiple comparisons using Bonferroni correction. Cohen’s *d* denotes effect size.
(DOCX)

S1 Fig Band-specific regional model fit. Separate regional regression models were applied to map MEG FC (AEC) to fMRI FC at each frequency band. Distributions of adjusted-*R*^2^ are depicted for band-specific regional model fits and for the multiband model fit obtained by the original analysis. The multilinear regional model that combines MEG connectivity at multiple rhythms to predict regional fMRI connectivity profiles performs better than the band-specific models. The data and code needed to generate this figure can be found in https://github.com/netneurolab/shafiei_megfmrimapping and https://zenodo.org/record/6728338. AEC, amplitude envelope correlation; FC, functional connectivity; fMRI, functional magnetic resonance imaging; MEG, magnetoencephalography.
(TIFF)

S2 Fig Structure–function coupling. Structure–function coupling was estimated as the Spearman rank correlation (*r*_*s*_) between regional structural and functional connectivity profiles [80]. The cross-modal *R*^2^ map (i.e., regional model fit) is then compared with the structure–function coupling across the cortex. The data and code needed to generate this figure can be found in https://github.com/netneurolab/shafiei_megfmrimapping and https://zenodo.org/record/6728338.
(TIFF)

S3 Fig Source localization error. MEG source localization error is estimated for (a) LCMV and (b) sLoreta source reconstruction solutions using CTFs [91–95]. CTF is used to calculate peak localization error of a given source *i* as the Euclidean distance between the peak location estimated for source *i* and the true source location *i* on the surface model [92,95]. No significant association is observed between the cross-modal correspondence *R*^2^ map and peak localization error for LCMV and sLoreta. The data and code needed to generate this figure can be found in https://github.com/netneurolab/shafiei_megfmrimapping and https://zenodo.org/record/6728338. CTF, cross-talk function; LCMV, linearly constrained minimum variance; MEG, magnetoencephalography; sLoreta, standardized low-resolution brain electromagnetic tomography.
(TIFF)

S4 Fig Signal-to-noise ratio. MEG SNR was estimated at the source level. Parcellated, group-average SNR map is depicted across the cortex. The cross-modal correspondence *R*^2^ map (i.e., regional model fit) is then compared with the SNR map. The data and code needed to generate this figure can be found in https://github.com/netneurolab/shafiei_megfmrimapping and https://zenodo.org/record/6728338. MEG, magnetoencephalography; SNR, signal-to-noise ratio.
(TIFF)

S5 Fig Pairwise similarity of band-limited MEG FC. Pearson correlation coefficient is calculated between upper triangles (i.e., values above diagonal) of band-limited MEG AEC FC to assess the pairwise similarity between MEG connectivity maps. The data and code needed to generate this figure can be found in https://github.com/netneurolab/shafiei_megfmrimapping and https://zenodo.org/record/6728338. AEC, amplitude envelope correlation; FC, functional connectivity; MEG, magnetoencephalography.
(TIFF)

## Abbreviations

AEC: amplitude envelope correlation
AHBA: Allen Human Brain Atlas
ANOVA: analysis of variance
ANT: Advanced Normalization Tool
CTF: cross-talk function
DWI: diffusion weighted imaging
ECG: electrocardiogram
ECoG: electrocorticography
EEG: electroencephalography
EOG: electrooculogram
FC: functional connectivity
FDR: false discovery rate
fMRI: functional magnetic resonance imaging
HCP: Human Connectome Project
LCMV: linearly constrained minimum variance
MEG: magnetoencephalography
NPY: Neuropeptide Y
NPY1R: Neuropeptide Y Receptor Y1
PLV: phase-locking value
sLoreta: standardized low-resolution brain electromagnetic tomography
SNR: signal-to-noise ratio
SSP: Signal–Space Projection
