# Supplementary material for: Human electromagnetic and haemodynamic networks systematically converge in unimodal cortex and diverge in transmodal cortex
Source: PLoS Biol. 2022 Aug 1;20(8):e3001735. doi: 10.1371/journal.pbio.3001735 (PMC9371256; doi:10.1371/journal.pbio.3001735)
Supplement: S1 Table — To quantitatively assess the differences in band-specific contributions to the cross-modal correspondence map, contributions estimated from dominance analysis were compared for all possible pairs of frequency bands using analysis of variance (ANOVA). All reported p-values are from two-tailed tests and are corrected for multiple comparisons using Bonferroni correction. Cohen’s d denotes effect size. (DOCX) [file pbio.3001735.s001.docx]

| **Band A** | **Band B** | **mean(A)** | **mean(B)** | **difference** | ***t*-value** | ***p*-value** | **Cohen’s *d*** |
| --- | --- | --- | --- | --- | --- | --- | --- |
| delta | theta | 15.06 | 19.87 | -4.81 | -5.26 | <0.0001 | -0.37 |
| delta | alpha | 15.06 | 18.79 | -3.72 | -4.07 | 0.00073 | -0.29 |
| delta | beta | 15.06 | 27.44 | -12.37 | -13.53 | <0.0001 | -0.96 |
| delta | lo-gamma | 15.06 | 11.28 | 3.78 | 4.14 | 0.00055 | 0.29 |
| delta | hi-gamma | 15.06 | 7.56 | 7.51 | 8.21 | <0.0001 | 0.58 |
| theta | alpha | 19.87 | 18.79 | 1.08 | 1.19 | 1 | 0.08 |
| theta | beta | 19.87 | 27.44 | -7.56 | -8.27 | <0.0001 | -0.58 |
| theta | lo-gamma | 19.87 | 11.28 | 8.59 | 9.39 | <0.0001 | 0.66 |
| theta | hi-gamma | 19.87 | 7.56 | 12.32 | 13.46 | <0.0001 | 0.95 |
| alpha | beta | 18.79 | 27.44 | -8.65 | -9.46 | <0.0001 | -0.69 |
| alpha | lo-gamma | 18.79 | 11.28 | 7.51 | 8.21 | <0.0001 | 0.58 |
| alpha | hi-gamma | 18.79 | 7.56 | 11.23 | 12.28 | <0.0001 | 0.87 |
| beta | lo-gamma | 27.44 | 11.28 | 16.16 | 17.66 | <0.0001 | 1.25 |
| beta | hi-gamma | 27.44 | 7.56 | 19.88 | 21.73 | <0.0001 | 1.54 |
| lo-gamma | hi-gamma | 11.28 | 7.56 | 3.72 | 4.07 | 0.00072 | 0.29 |
