# Supplementary figures and images for: Human electromagnetic and haemodynamic networks systematically converge in unimodal cortex and diverge in transmodal cortex
Source: PLoS Biol. 2022 Aug 1;20(8):e3001735. doi: 10.1371/journal.pbio.3001735 (PMC9371256; doi:10.1371/journal.pbio.3001735)

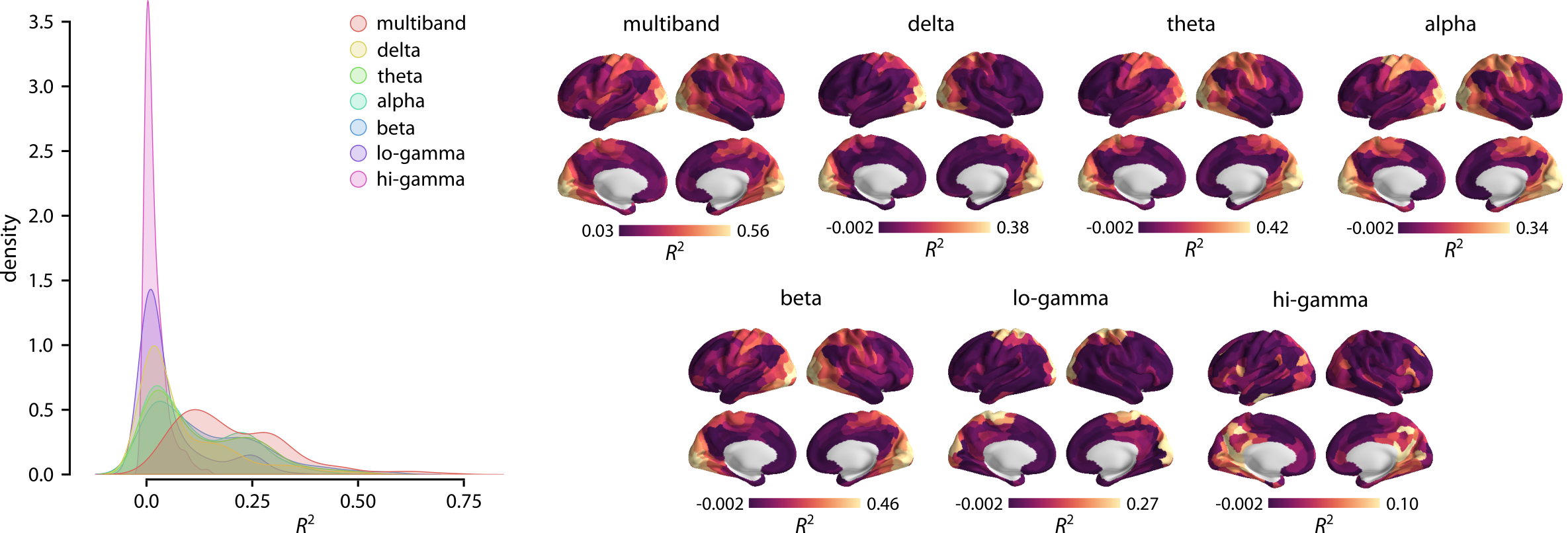

Supplement: S1 Fig — Separate regional regression models were applied to map MEG FC (AEC) to fMRI FC at each frequency band. Distributions of adjusted-R2 are depicted for band-specific regional model fits and for the multiband model fit obtained by the original analysis. The multilinear regional model that combines MEG connectivity at multiple rhythms to predict regional fMRI connectivity profiles performs better than the band-specific models. The data and code needed to generate this figure can be found in https://github.com/netneurolab/shafiei_megfmrimapping and https://zenodo.org/record/6728338. AEC, amplitude envelope correlation; FC, functional connectivity; fMRI, functional magnetic resonance imaging; MEG, magnetoencephalography. (TIFF) [file pbio.3001735.s002.tiff]

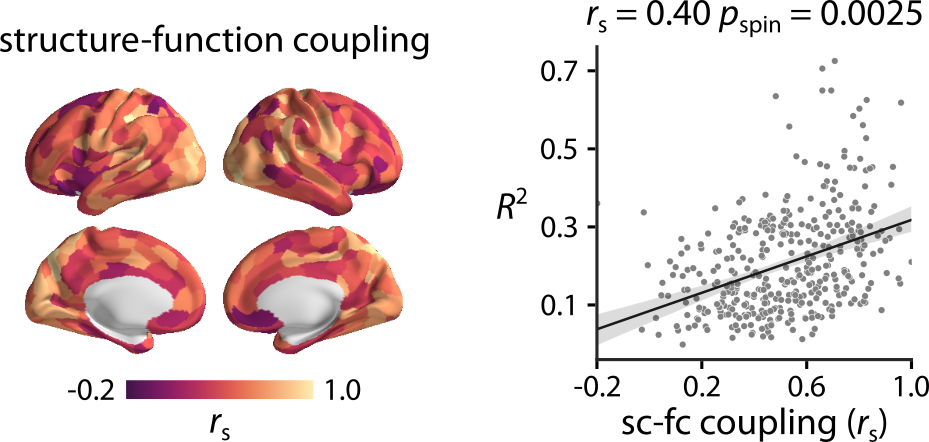

Supplement: S2 Fig — Structure–function coupling was estimated as the Spearman rank correlation (rs) between regional structural and functional connectivity profiles [80]. The cross-modal R2 map (i.e., regional model fit) is then compared with the structure–function coupling across the cortex. The data and code needed to generate this figure can be found in https://github.com/netneurolab/shafiei_megfmrimapping and https://zenodo.org/record/6728338. (TIFF) [file pbio.3001735.s003.tiff]

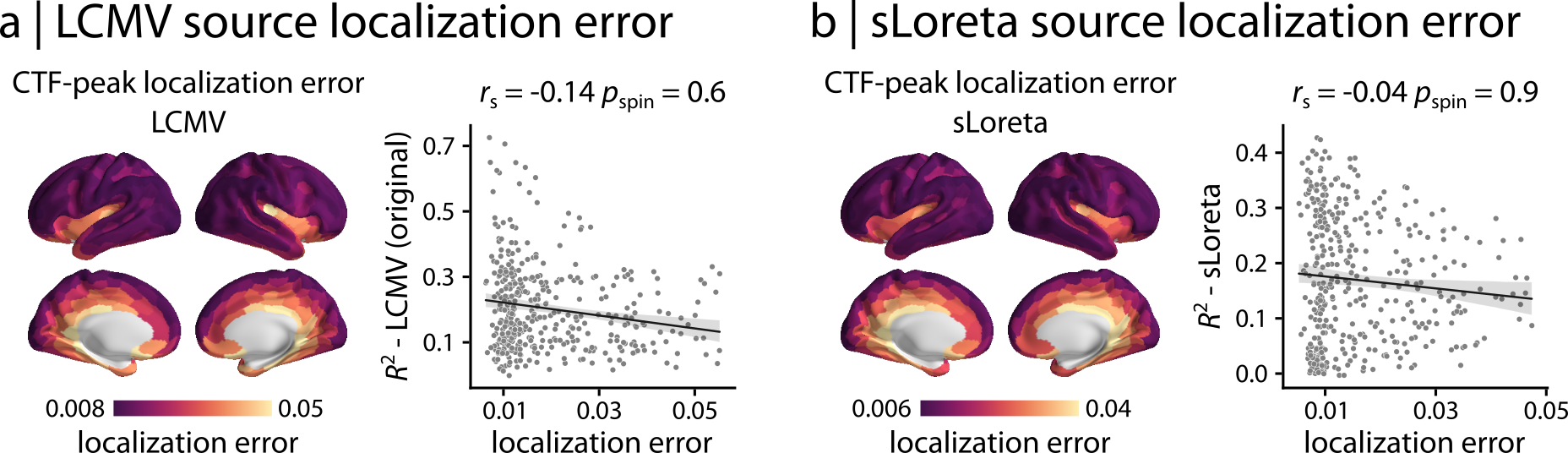

Supplement: S3 Fig — MEG source localization error is estimated for (a) LCMV and (b) sLoreta source reconstruction solutions using CTFs [91–95]. CTF is used to calculate peak localization error of a given source i as the Euclidean distance between the peak location estimated for source i and the true source location i on the surface model [92,95]. No significant association is observed between the cross-modal correspondence R2 map and peak localization error for LCMV and sLoreta. The data and code needed to generate this figure can be found in https://github.com/netneurolab/shafiei_megfmrimapping and https://zenodo.org/record/6728338. CTF, cross-talk function; LCMV, linearly constrained minimum variance; MEG, magnetoencephalography; sLoreta, standardized low-resolution brain electromagnetic tomography. (TIFF) [file pbio.3001735.s004.tiff]

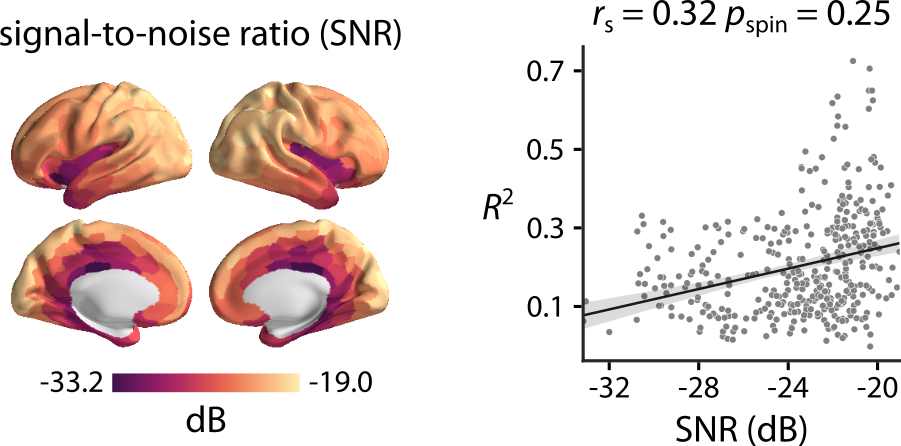

Supplement: S4 Fig — MEG SNR was estimated at the source level. Parcellated, group-average SNR map is depicted across the cortex. The cross-modal correspondence R2 map (i.e., regional model fit) is then compared with the SNR map. The data and code needed to generate this figure can be found in https://github.com/netneurolab/shafiei_megfmrimapping and https://zenodo.org/record/6728338. MEG, magnetoencephalography; SNR, signal-to-noise ratio. (TIFF) [file pbio.3001735.s005.tiff]

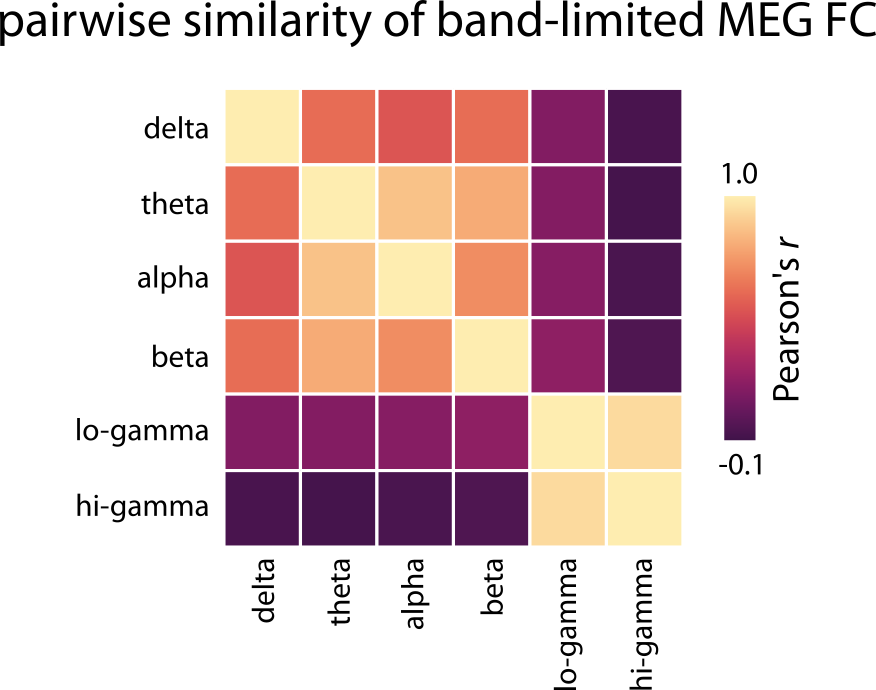

Supplement: S5 Fig — Pearson correlation coefficient is calculated between upper triangles (i.e., values above diagonal) of band-limited MEG AEC FC to assess the pairwise similarity between MEG connectivity maps. The data and code needed to generate this figure can be found in https://github.com/netneurolab/shafiei_megfmrimapping and https://zenodo.org/record/6728338. AEC, amplitude envelope correlation; FC, functional connectivity; MEG, magnetoencephalography. (TIFF) [file pbio.3001735.s006.tiff]
